# Genkwanin nanosuspensions: a novel and potential antitumor drug in breast carcinoma therapy

**DOI:** 10.1080/10717544.2017.1384519

**Published:** 2017-09-29

**Authors:** Yijing Li, Jingyi Hong, Haowen Li, Xiaoyu Qi, Yifei Guo, Meihua Han, Xiangtao Wang

**Affiliations:** a Institute of Medicinal Plant Development, Chinese Academy of Medical Sciences & Peking Union Medical College, Beijing, PR China;; b Institute of Allergy and Immunology, Shenzhen University School of Medicine, Shenzhen, Guangdong Province, PR China;; c School of Pharmacy, Heilongjiang University of Chinese Medicine, Harbin, PR China

**Keywords:** Genkwanin, nanosuspensions, TPGS, cytotoxicity, antitumor activity

## Abstract

Recently, genkwanin (GKA) has been shown to display *in vitro* antitumor activity against some cancer cells, but its poor solubility restricted the *in vivo* study and further investigation of its antitumor therapeutic efficacy. In this paper, genkwanin nanosuspensions (GKA-NSps) were successfully prepared using D-alpha tocopherol acid polyethylene glycol succinate (TPGS) as a stabilizer using the precipitation-homogenization method. The obtained GKA-NSps had an average particle size of 183.1 ± 4.4 nm, a PDI value of 0.16 ± 0.07, a zeta potential of −16.2 ± 0.1 mV, and a drug loading content of 49.36 ± 0.14%. GKA-NSps showed spherical morphology and very good stability in normal saline, phosphate buffer saline (PBS, pH 7.4), 5% glucose, artificial gastric juice, artificial intestinal juice and plasma; thus, it is suitable for both oral and intravenous administration. The resultant GKA-NSps displayed sustained drug release behavior and stronger *in vitro* cytotoxicity against 4T1, MCF-7, MDA-MB-453, HeLa, HepG2, BT474, and A549 cells than free GKA. The *in vivo* study in MCF-7 tumor-bearing nude mice indicated that GKA-NSps (60 mg/kg, i.v.) achieved similar therapeutic efficacy as PTX injection (8 mg/kg, i.v.) (62.09% vs. 61.27%), while the minimal lethal dose was more than 320 mg/kg, indicating good safety. By using nanotechnology, our study suggested that some antitumor flavonoids of low potency, such as GKA, are promising as safe but effective anticancer drugs.

## Introduction

Flavonoids are a large type of nature-resourced compounds that are widespread in vegetable, fruits and medicinal plants. Flavonoids have displayed extensive biological activities including immunoregulation, antioxidation, neuroprotection, antibacterial and so on (Silvan et al., 2011; Yang et al., 2015), most of which are beneficial to human health. Recently, many studies have shown that some natural flavonoids have a certain resistance against breast cancer, lung cancer, prostate cancer, liver cancer, gastric cancer and other tumors. However, in most cases, the antitumor potency was low (Wang et al., 2005; Batra & Sharma, 2013; Lin et al., 2013; Mateen et al., 2013; Moon et al., 2013; Pandurangan et al., 2013; Feng et al., 2014; Mahmoud et al., 2014; Yi et al., 2014; Yiannakopoulou, 2014).

Genkwanin (GKA) is a typical bioactive non-glycosylated flavonoid isolated from Genkwa Flos (*Daphne Genkwa* Sieb. et Zucc.), rosemary (*Rosmarinus officinalis* L.) (Altinier et al., 2007) and the leaves of *Cistus laurifolius* L. (Sadhu et al., 2006). It was defined as the representative marker for quality control of related traditional Chinese prescriptions (Li et al., 2010). Previous evidence indicated that GKA has various pharmacological effects including antitussive, expectorant, anti-inflammatory (Gao et al., 2014), anti-bacterial (Cottiglia et al., 2001; Martini et al., 2004), antiplasmodial (Kraft et al., 2003), chemopreventive (Suh et al., 1995), and radical scavenging (Kim et al., 2004) activities. In addition, it has also been reported that GKA has a certain anti-tumor effect. The results of Wang et al. (2015) indicated that GKA inhibited tumor cell proliferation partly by strengthening host immunity and reducing the levels of inflammatory cytokine. Androutsopoulos et al. (2009) reported that GKA inhibited the *in vitro* proliferation of MDA-MB-468 breast cancer cells at micromolar concentrations. GKA also exhibited anti-proliferative activity against B16F10 melanoma cells (Nasr Bouzaiene et al., 2016) and cotton-pellet-induced granuloma (Pelzer et al., 1998). However, GKA is not very water-soluble (<1 μg/mL), and this limits its further *in vivo* research and clinical application. In Wang’s study, genkwanin was evenly mixed with a high-fat chow, and the daily oral genkwanin dose was calculated based on the amount of chow consumption (Wang et al., 2015). In other *in vivo* studies, which have been reported thus far (Song et al., 2013), the authors failed to describe how GKA was orally or intravenously or intraperitoneally administered. Up to now, there has been no dosage form study reported on GKA.

Nanosuspensions (Li et al., 2014), as a new type of nanoscale drug delivery system, have attracted significant attention due to their ability to increase the solubility of insoluble drugs (Muller et al., 2001) and their suitability for various administration routes. To overcome the solubility problem and further investigate the potential of GKA as a drug for application in clinic, GKA was prepared into nanosuspensions using the antisolvent precipitation combined with the ultrasonication method (Das & Suresh, 2011; Han et al., 2014). Surprisingly, GKA nanosuspensions significantly enhanced the *in vitro* anti-tumor activity against all the tested human tumor cell lines. The *in vivo* examination on MCF-7 tumor-bearing mice displayed that 5 doses of the intravenous administration of GKA-NSps (60 mg/kg) every other day for 10 days achieved the inhibition rate of 62%, which is similar to that achieved via 8 mg/kg of PTX injections (61%). These demonstrated nanosuspensions can solve the solubility problem of GKA and also significantly improve its antitumor efficacy.

As far as we know, this is the first study on the dosage form of GKA, and the first report on the anti-tumor research using GKA nanoparticles. This paper provided an experimental basis for the future investigation of genkwanin in the treatment of tumor and provided a possible approach for other antitumor flavonoids to become safe and effective drugs.

## Materials and methods

### Materials

Genkwanin with more than 98% purity was purchased from Chengdu Herbpurify Co. Ltd. (Chengdu, China). Methoxy (poly ethylene glycol) 2000–poly (ε-caprolactone) 2000 (mPEG2000–PCL2000) was obtained from Jinan Daigang Biomaterial Co. Ltd. (Jinan, China). D-alpha tocopherol acid polyethylene glycol succinate (TPGS) was supplied by Xi’an Healthful Biotechnology Co. Ltd. (Xi’an, China). Pluronic F-127 (F-127) was purchased from Sigma Aldrich (St Louis, MO). Paclitaxel (PTX) injections were obtained from the Beijing union pharmaceutical factory (Beijing, China). The 3-(4,5-dimethylthiazol-2-yl)-2,5-diphenyltetrazolium bromide (MTT) was provided by Sigma-Aldrich Co., (St Louis, MO). Methanol (HPLC grade) was purchased from Fisher Scientific (Pittsburgh, PA). The water used in the experiments was deionized. All other organic solvents and chemicals were of the highest commercial level available.

### Cell lines and animals

The 4T1 (murine mammary carcinoma), MCF-7 (breast carcinoma), HeLa (cervix carcinoma), HepG2 (hepatic carcinoma), MDA-MB-453 (breast carcinoma), BT474 (breast carcinoma), A549 (pulmonary carcinoma), and HUVEC (human umbilical vein endothelial cell) cell lines were purchased from Cell Culture Center, Institute of Basic Medical Sciences (Beijing, China). The 4T1 cells and BT474 cells grew in a Roswell Park Memorial Institute 1640 medium (RPMI 1640, Thermo Fisher Scientific). MCF-7 cells, HeLa cells and A549 cells were cultured using Dulbecco’s modified Eagle’s medium (DMEM, Thermo Fisher Scientific). HepG2 cells grew in a Minimum Essential medium (MEM, Thermo Fisher Scientific). MDA-MB-453 cells were cultivated using Leibovitz’s 15 medium (L-15, Thermo Fisher Scientific). HUVECs were cultivated using Ham’s F12 medium (F12, Thermo Fisher Scientific). Different cells were cultured in different media containing 10% fetal calf serum (Thermo Fisher Scientific), penicillin (100 U/mL), and streptomycin (100 U/mL) at 37 °C with 5% CO_2_ (Sanyo, Osaka, Japan).

Female NU/NU nude mice (6–8 weeks old, 20 ± 2 g) were supplied by Vital River Laboratory Animal Technology Co., Ltd. (Beijing, China). The animals were acclimated at a relative humidity of 70%±5% and 25 °C under 12 h light–dark cycle conditions for 1 week before the experiments. All animal experiments were performed based on the Guidelines for Ethical and Regulatory for Animal Experiments as defined by Institute of Medicinal Plant Development (IMPLAD), China.

### Preparation of genkwanin nanosuspensions (GKA-NSps)

GKA-NSps were prepared via the antisolvent precipitation method. Briefly, the GKA bulk powder and stabilizer at different weight ratios were co-dissolved in dimethyl formamide (DMF) to form a mixed organic solution containing 20 mg/mL of drug. Then, 0.2 mL of the mixed solution was slowly injected into 4 mL of deionized water at 25 ± 2 °C under 250 W ultrasonication (Ultrasonic cleaner, Kun Shan Ultrasonic Instruments Co., Ltd., China). Subsequently, the resultant suspensions were centrifuged at 13,000 rpm for 20 min using a high-speed centrifuge (Sigma-Aldrich Co., Ltd., Germany), and the sediment was re-dispersed into deionized water using ultrasonication at 250 W for 20 min, followed by homogenization for 10 times under 1500 bar at 25 °C to obtain GKA-NSps.

TPGS, mPEG2000-PCL2000, and F-127 were respectively tried as a stabilizer in this study to prepare GKA-NSps (drug: stabilizer = 1:1, weight ratio). Then, different drug-stabilizer ratios were tried for the best stabilizing effect.

### Physicochemical characterizations of GKA-NSps

#### Particle size of GKA-NSps

The mean particle size, the polydispersity index (PDI), and the zeta potential of GKA-NSps were determined via dynamic light scattering (Zetasizer Nano ZS, Malvern Instruments, UK) at 25 °C. Each sample was measured in triplicate, and all data were expressed as the mean ± standard deviation (SD). GKA-NSps were kept at 4 °C, and the particle size of GKA-NSps during storage was monitored to evaluate their physical stability.

#### Morphology of GKA-NSps

The morphological evaluation of GKA-NSps was conducted using a JEM-1400 transmission electron microscope (TEM, JEOL, Tokyo, Japan). A drop of GKA-NSps was dropped on the surface of the 300-mesh copper grid, air-dried, and dyed with 2% (w/v) uranyl acetate for observation under electron microscope.

#### X-ray diffraction (XRD) measurements

An X-ray diffractometer (DX-2700, China) was used to detect X-ray powder diffraction with a generator set at 100 mA and 40 kV. The samples were scanned over an angular range of 3–80° of 2 θ, with a step size of 0.02° and a count time of 3 s per step. The samples were rotated at 30 rpm during the analyses.

#### Differential scanning calorimetry (DSC) characterization

DSC thermal profiles of the powder samples were tested using a differential scanning calorimeter (Q200, TA Instruments, New Castle, DE). The samples of approximately 5 mg were placed in standard aluminum pans, sealed with a lid and measured from 0 to 500 °C at a scanning rate of 10 °C/min under nitrogen atmosphere.

#### Thermogravimetric analysis

Thermogravimetric analysis of the powder samples were tested using a thermogravimetric apparatus (Q 50, TA Instruments, New Castle, DE). The samples were placed in standard aluminum pans and measured from 0 to 500 °C at a rate of 10 °C/min under nitrogen atmosphere.

#### Drug loading of GKA-NSps

Drug loading content (DLC) of GKA-NSps was determined as follows. Briefly, a certain amount of lyophilized GKA-NSps was dissolved in a certain amount of methanol. The concentration of GKA in the solution was determined using HPLC. The DLC of GKA-NSps was calculated according to the following Equation (1):

(1) DLC (%)=V·C/W × 100

where V is the volume of methanol, C is the concentration of GKA, and W is the weight of lyophilized powder of GKA-NSps.

### High-performance liquid chromatography analysis of GKA

The concentration of GKA was determined using a high-performance liquid chromatography instrument (HPLC, DIONEX Ultimate 3000, Germering, Germany). Solvent delivery system was equipped with an autosampler and a column heater. Chromatographic separations were performed using a Symmetry C18 column (4.6 mm × 250 mm, 5 μm; Dr. Maisch GmbH, Germany) at 25 °C. The mobile phase was composed of methanol and water (85:15, v/v) and ran at a flow rate of 1.0 mL/min. The detection wavelength of UV detector was set at 337 nm. The standard curve equation was Y = 1.5214X + 0.2021 (here, Y is the area of GKA peak; X is the concentration of GKA; linear range is 0.5–50 μg/mL) and the correlation coefficient was 0.9995.

### The size change of GKA-NSps in various physiological media

Here, physiological media include normal saline, phosphate buffer saline (PBS, pH 7.4), isotonic glucose (5% Glucose), plasma, simulated gastric fluid (containing 1.0% pepsinum in 1 mol/L diluted HCl), and simulated intestinal fluid containing 1.0% pancreatin in pH 6.8 PBS (0.01 M). To study whether physiological media can interact with GKA-NSps and then induce nanoparticles aggregation, *in vitro* stability test was carried out as follows. GKA-NSps were respectively mixed with 1.8% NaCl, 2 × PBS, and 10% glucose (1:1, v/v), followed by incubation at 37 °C and measurement of particle size and distribution at specific time intervals; to evaluate the suitability of GKA-NSps for intravenous injection and oral administration, GKA-NSps were mixed (1:4, v/v) with rat plasma, simulated gastric fluid, and simulated intestinal fluid, respectively, and then incubated at 37 °C and measured for particle size and distribution at specific time intervals. Each experiment was repeated three times.

### Hemolytic assay

To conduct a preliminary investigation of the safety of the obtained GKA-NSps for intravenous administration, healthy rat red blood cells (RBCs) were used to examine the hemolysis of GKA-NSps. Fresh rat blood was centrifuged at 5000 rpm for 10 min to remove the supernatant, successively washed with normal saline and diluted to 4% (v/v). The obtained RBC suspension was mixed (1:1, v/v) with deionized water (as a positive control) and 0.9% NaCl (as a negative control). Different concentrations of GKA-NSps were adjusted to isotonic solutions and then mixed (1:1, v/v) with 4% red blood cell suspensions (as experimental groups). In addition, different concentrations of GKA-NSps were mixed (1:1, v/v) with deionized water (as a blank control). The mixtures were incubated at 37 °C for 4 h and then centrifuged at 5000 rpm for 5 min. The absorbance of the supernatant was detected at 540 nm using an ELISA plate reader (Biotek, Winooski, VT). The hemolysis rate was calculated according to the following Equation (2):

(2) $$\text{Hemolytic rate }\left(\%\right)=\left({\text{A}}_{\text{sample}}–{\text{ A}}_{\text{negative}}–{\text{ A}}_{\text{blank}}\right)/\left({\text{A}}_{\text{positive}}–{\text{ A}}_{\text{negative}}\right)\times \text{1}00$$

where A_sample_ is the absorbance value of the experimental group, A_negative_ is the absorbance value of the negative control, A_positive_ is the absorbance value of the positive control, and A_blank_ is the absorbance value of the blank control. Each sample was carried out in triplicate.

### *In vitro* drug release behavior

The dialysis bag diffusion method was adopted to measure the *in vitro* drug release of GKA-NSps. GKA-NSps (4 mL, 100 μg/mL) were placed in dialysis tubing molecular weight cut off (MWCO): 8000–14000, Sigma. Then, the dialysis tubes were immersed in 1 L of a pH 7.4 phosphate buffer solution containing 5% (w/v) albumin bovine V (BSA, Beijing Biotopped Science & Technology CO., Ltd.) and incubated at 37 °C with constant stirring (100 rpm). In total, 50 μL of internal dialysate was withdrawn from the dialysis tubing at specific time intervals, and the tubes were replenished with the same volume of fresh release medium. The external release medium was replaced with new one every 24 h. The cumulative release of GKA-NSps was calculated according to the reduction of GKA-NSps inside the dialysis tubing. Briefly, 50 μL of internal dialysate was mixed with 450 μL of methanol for disintegration of GKA-NSps and drug release, and then centrifuged (13,000 rpm, 20 min); the supernatant was analyzed using HCPT for GKA concentration. The above experiments were performed in triplicate.

### *In vitro* cytotoxicity assay

The cytotoxicity of GKA-NSps was measured using the MTT assay. The assay was performed on 4T1, MCF-7, MDA-MB-453, BT474, HeLa, HepG2, A549 and HUVECs. Typically, 150 μL of cells (at a density of 5.3 × 10^4^ cells/mL) were seeded in each well of 96-well plates and incubated overnight at 37 °C in 5% CO_2_. Then, the cells were exposed to different concentrations of GKA-NSps or free GKA solution dissolved in dimethylsulfoxide (DMSO), final concentration of DMSO ≤0.1% diluted with a culture medium for 48 h using TPGS and DMSO as a bank control. After that, the cells were exposed to 20 μL of an MTT solution (5 mg/mL in PBS) for 4 h. Subsequently, the medium was removed, and 150 μL of DMSO was added to each well to dissolve the formazan crystals. The maximum absorbance was detected at 570 nm using an ELISA plate reader (Biotek, Winooski, VT). The cell viability was calculated according to the following Equation (3):

(3) $$\text{Cell viability }\left(\%\right)={\text{OD}}_{\text{e}}/{\text{OD}}_{\text{c}}\times \text{1}00$$

where OD_e_ is the mean optical density of the experimental group, and OD_c_ is the mean optical density of the control group.

The half maximal inhibitory concentration (IC50) of GKA-NSps was calculated using the GraphPad Prism software, Version 5 (GraphPad Software, Inc., La Jolla, CA) via the sigmoidal dose–response variable curve-fitting method.

### *In vivo* antitumor activity in MCF-7 tumor-bearing mice

*In vivo* anticancer effect of GKA-NSps was performed on MCF-7 tumor-bearing NU/NU mice. Briefly, female nude mice were inoculated subcutaneously with 0.2 mL of MCF-7 cells suspended in a culture medium at a density of 4.0 × 10^7^ cells/mL in the right armpit under sterile conditions. When the volume of tumor reached 100 mm^3^, the MCF-7 tumor-bearing mice were randomly divided into 7 groups (7 mice per group). The mice were injected via the lateral tail vein every two days with normal saline (as a negative control), paclitaxel injection (8 mg/kg, as a positive control), free TPGS (60 mg/kg), GKA-NSps (20 mg/kg), GKA-NSps (40 mg/kg), and GKA-NSps (60 mg/kg) or given by gavage once a day with GKA-NSps (60 mg/kg), respectively. During the entire administration process, the volume of tumors and the body weight were measured every 2 days. The mice were killed via cervical vertebra dislocation and dissected on the 10th day of treatment. The tumors were excised and weighed. Tumor volume was calculated using Equation (4):

(4) $$\text{V}=\;\left(\text{a}\cdot {\text{b}}^{\text{2}}\right)/\text{2}$$

where V is the tumor volume, a is the length of the major axis, and b is the length of the minor axis.

The tumor inhibition rate (TIR) was calculated using the following Equation (5):

(5) $$\text{TIR }\left(\%\right)=\left(\text{1 }–{\text{ W}}_{\text{e}}/{\text{W}}_{\text{n}}\right)\times \text{1}00$$

where W_e_ is the mean tumor weight of the experimental group, and W_n_ is the mean tumor weight of the negative control group.

### Statistical analysis

The statistical analysis among the different groups was carried out using the IBM SPSS Statistics software, Version 19 (IBM Corporation, Armonk, NY). Comparisons of IC50 between GKA-NSps and the free GKA DMSO solution were performed using the independent samples *T* test. Comparison of the tumor inhibition rate was based on a one-way analysis of variance. The *p* value that was .05 or less was considered statistically significant.

## Results and discussion

### Preparation and characterizations of GKA-NSps

Stabilizer plays a very important role in the preparation of ideal nanosuspensions using a bottom-up method. It can help form a small size of nanosuspensions via timely attachment at the surface of drug nanocrystals to terminate crystal growth. It can also increase the stability of the obtained nanoparticles through the formation of a flexible and hydrophilic hydration shell around the nanoparticles. However, there is still not a fully developed theory to guide how to select the optimal stabilizer for a specific drug molecule. In this study, TPGS, PEG2000-PCL2000, and F-127 were tried as stabilizers to prepare GKA-NSps, initially at the drug-stabilizer ratio of 1:1 (weight ratio), aimed at small mean diameter (less than 200 nm) and being stable for at least 3 days. Anti-solvent nanoprecipitation was tried. It was determined that nanoprecipitation alone led to a large size of GKA nanosuspensions (>500 nm), thus homogenization was further employed to reduce the particle size. As seen in Table 1, among the three adjutants, TPGS as a stabilizer resulted in the smallest mean diameter, the narrowest size distribution, the highest zeta potential and the best storage stability (Figure S1). Therefore, TPGS was selected as a stabilizer to prepare GKA-NSps in the subsequent study.

**Table 1.** Characterization of GKA-NSps prepared using different stabilizer and ratios of GKA/TPGS (mean ± SD, *n* = 3).

Stabilizer	GKA: stabilizer	Size(nm)	PDI	Zeta potential (mV)	4 °C storage timewith similar size (h)	DLC (%)
PEG_2000_–PCL_2000_	1:1	184.7 ± 5.4	0.18 ± 0.02	−12.3 ± 1.2	60	–^a^
F-127	1:1	222.7 ± 10.9	0.22 ± 0.03	−6.6 ± 2.0	24	–^a^
TPGS	1:1	183.1 ± 4.4	0.16 ± 0.07	−16.2 ± 0.1	>1440	49.36 ± 0.14
TPGS	2:1	248.0 ± 8.3	0.20 ± 0.01	−15.2 ± 0.5	>576	61.29 ± 0.06
TPGS	3:1	332.3 ± 5.6	0.25 ± 0.03	−14.2 ± 0.4	>576	69.16 ± 0.21

a The data are not tested.

Furthermore, the effect of GKA/stabilizer ratios on the properties of nanosuspensions was investigated. As shown in Table 1, the drug loading, particle size, and PDI increased with the increase of GKA/stabilizer ratio, whereas the zeta potential (absolute value) decreased accordingly. In consideration of size, stability and drug-loading content, GKA/stabilizer ratio (1/1) was chose for further applications.

**Figure 1.**
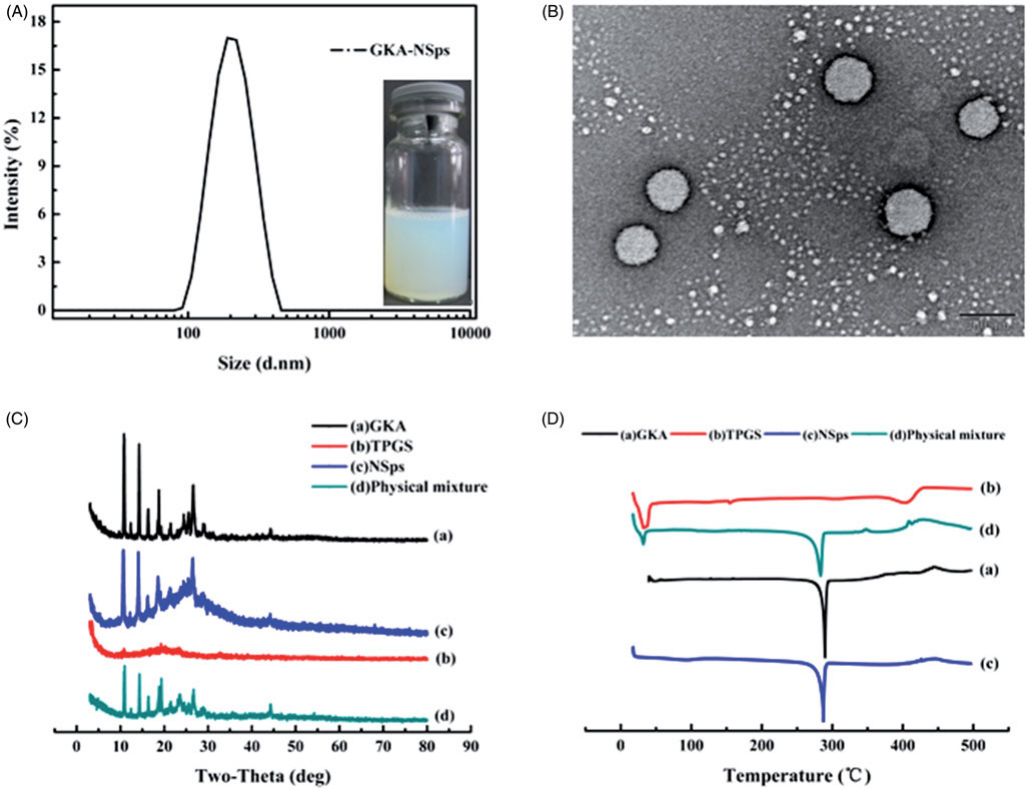
Preparation and characterization of GKA-NSps. (A) Particle size distribution and photograph of GKA-NSps; (B) TEM image of GKA-NSps; (C) XRD patterns of the GKA bulk powder, stabilizer (TPGS), GKA-NSps, and the physical mixture of GKA bulk powder and TPGS; (D) Differential scanning calorimetry thermograms of the GKA bulk powder, stabilizer (TPGS), GKA-NSps, and the physical mixture of GKA bulk powder and TPGS.

The obtained GKA-NSps had a mean particle size of 183.1 ± 4.35 nm, PDI value of 0.16 ± 0.067 (Figure 1(A)) and DLC of 49.36 ± 0.14%. A small PDI value (<0.2) indicated that the size distribution of GKA-NSps was narrow. The highest concentration of GKA in the nanosuspensions reached 32 mg/kg, over 30,000 times higher compared to its original solubility at 25 °C (0.1 μg/mL) (Gong et al., 2013). GKA-NSps showed clear colloidal solution with light blue opalescence (Figure 1(A)). However, when bulk GKA was dispersed in water at the same concentration, it formed a turbid suspension with a sediment at the bottle bottom (Figure S2). Thus, the solubility problem of GKA is well solved.

Transmission electron microscopy image (Figure 1(B)) revealed that GKA-NSps were spherical and regular in shape. The diameter observed using TEM was in good agreement with the results determined via dynamic light scattering.

XRD patterns of the GKA bulk powder, stabilizer (TPGS), GKA-NSps, and the physical mixture of GKA bulk powder and TPGS were measured under the same condition, as shown in Figure 1(C). The diffractogram of GKA bulk powder showed sharp and intense diffraction peaks of crystallinity, indicating a crystalline structure for GKA. The diffraction pattern of the lyophilized GKA-NSps was consistent with that of the bulk powders and the physical mixture, revealing that GKA also existed in the crystalline form in the obtained nanosuspensions.

The DSC investigation (Figure 1(D)) showed similar results. Both the GKA bulk powder and the physical mixture exhibited a sharp endothermic peak at approximately 289.9 °C, which is the melting point of GKA. GKA-NSps showed an identical melting temperature and melting endothermic peak, suggesting that the crystalline form of GKA had not changed during the preparation of GKA-NSps.

The thermogravimetric analysis results are shown in Figure S3. No mass loss can be observed until degradation at approximately 350 °C for the GKA bulk powder, GKA-NSps, and the physical mixture, demonstrating that all of the three samples were very dry with no water or solvent residual. As a result, the above DSC result has been proven to be reliable.

### Stability in physiological media and hemolysis ofGKA-NSps

**Figure 2.**
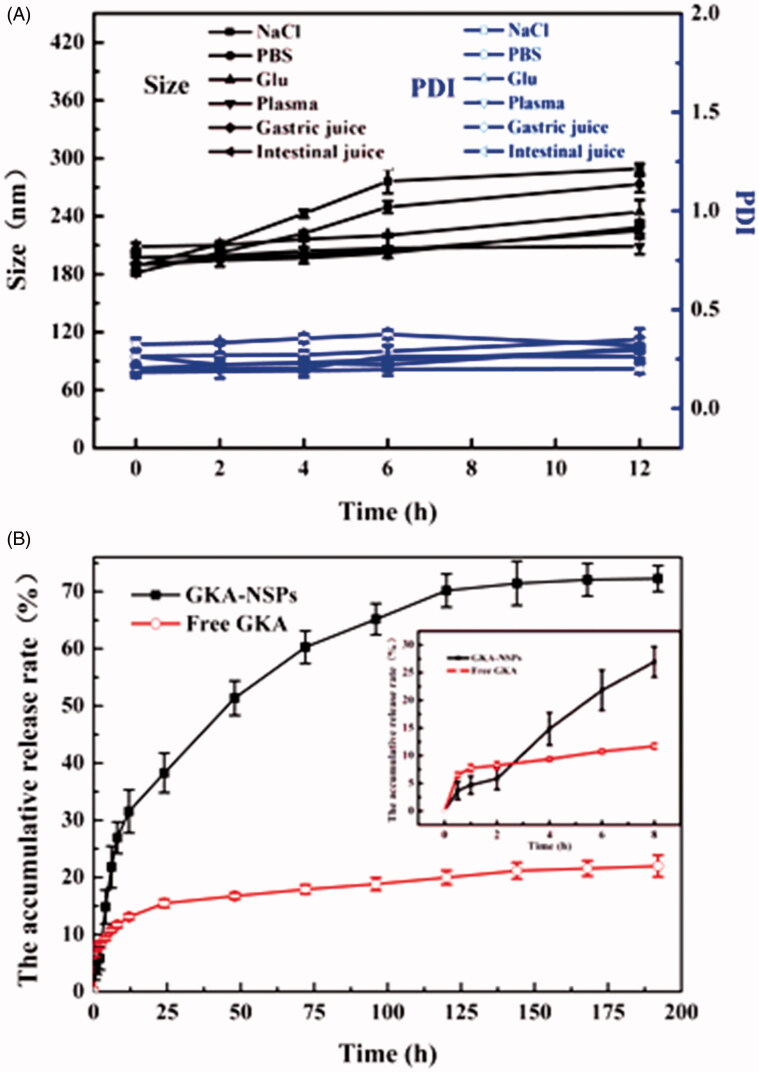
Stability and *in vitro* drug release profiles of GKA-NSps. (A) Size and PDI changes of the GKA-NSps after incubation with various physiological media at 37 °C for 12 h; (B) The *in vitro* drug release profiles of GKA-NSps and GKA coarse suspensions in pH 7.4 PBS containing 5% (w/v) BSA at 37 °C (mean ± SD, *n* = 3).

GKA-NSps were stable in physiological saline, PBS, and isotonic glucose after incubation at 37 °C for 12 h despite the slightly larger diameter than in deionized water (Figure 2(A)). There were no aggregation and no significant particle size enlargement during the incubation of GKA-NSps with an artificial intestinal fluid or gastric fluid, suggesting the suitability of GKA-NSps for oral drug delivery.

There are various enzymes and serum albumins in plasma that can be absorbed on the surface of nanoparticles and in some cases resulting in aggregation or blood blockage. Fortunately, GKA-NSps maintained their size during 12 h of incubation with rat plasma, indicating their good plasma stability (Figure 2(A)). GKA-NSps showed no hemolysis below 6 mg/mL (Figure S4). This meant that GKA-NSps also meet the demand of intravenous injection.

### *In vitro* drug release behavior

The *in vitro* drug release behavior of GKA-NSps was investigated employing the dialysis bag diffusion method. GKA-NSps in PBS containing 0.5% Tween 80 or 5% lauryl sodium sulfate (SDS) was not stable, with size enlargement to micrometer level or even precipitation. Thus, PBS containing 5% (w/v) BSA was chosen as the release medium. The cumulative dissolution profiles are shown in Figure 2(B). Only 22.0% of drug was released from GKA coarse suspensions (made by directly dispersing GKA bulk powder in deionized water). However, for GKA-NSps, 28.9% of the drug was released in the first 8 h, followed by a steady drug release up to 70.2% at 120 h, and then a slow release until 192 h. The much higher cumulative drug release of GKA-NSps was attributed to their small particle size and greatly enhanced surface area, which has been widely reported (Han et al., 2013; Hong et al., 2016).

### *In vitro* cytotoxicity

**Figure 3.**
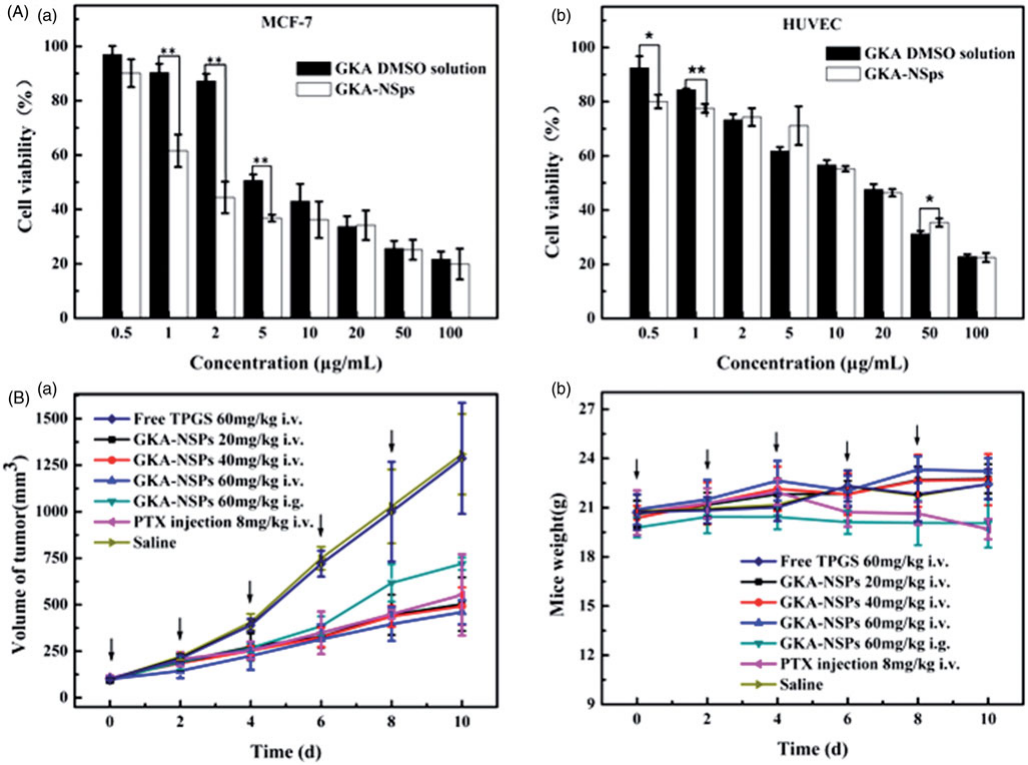
*In vitro* cytotoxicity studies and *in vivo* antitumor activity against MCF-7 tumor-bearing mice. (A) Cytotoxicity of GKA-NSps and GKA DMSO solution against MCF-7 cells (a) and normal HUVECs (b) after 48 h of incubation (mean ± SD, *p < .05, **p < .01); (B) (a) The growth of tumor volume with administration in each group; (b)The body weight change of mice over time (mean ± SD, *n* = 7, ↓represents administrating of drugs).

Cytotoxicity of GKA-NSps and GKA/DMSO solution against 4T1, MCF-7, MDA-MB-453, BT474, HeLa, HepG2, A549, and HUVECs after 48 h of incubation was assessed using the MTT assay. Both GKA-NSps and GKA solution inhibited the proliferation of these cells in a dose-dependent manner, but GKA-NSps exhibited a significantly higher cytotoxicity than GKA solution against all examined cancer cell lines Figure 3(A) (a), Figure S5, with IC50 values reduced to approximately 1/3 or more (2.86 ± 0.55 vs. 9.42 ± 2.14 for MCF-7, 3.909 ± 0.52 vs. 14.2 ± 2.23 for A549, 7.862 ± 1.73 vs. 25.16 ± 3.12 for HepG2, 12.31 ± 2.17 vs. >100 for HeLa, *p* < .05 in all cases, Table 2). This type of enhancement of *in vitro* antitumor activity through drug encapsulation into nanoparticles has been widely reported. It was reported that Fernandez-Urrusuno and Dong proved that tumor cells can conduct nonspecific adsorption to drug-loading nanoparticles and enhance their internalization via endocytosis and other mechanisms (Fernandez-Urrusuno et al., 1996; Dong et al., 2016).

**Table 2.** IC50 values of GKA-NSps and GKA solution against different tumor cell lines and HUVECs after incubation for 48 h (mean ± SD).

IC50(μg/mL) of cancer cells	GKA-NSps	GKA DMSO solution
4T1	42.09 ± 8.83**	>100
MCF-7	2.86 ± 0.55**	9.42 ± 2.14
MDA-MB-453	52.87 ± 9.03**	>100
HeLa	7.86 ± 1.73**	25.16 ± 3.12
HepG2	12.31 ± 2.17**	>100
BT474	>100	>100
A549	3.91 ± 0.52**	14.20 ± 2.23
HUVEC	14.82 ± 2.84	14.15 ± 1.62

**p < .01 vs. GKA solution.

Meanwhile, cytotoxicity of TPGS and DMSO was investigated as a control (Figure S6). It was shown that after 48 h of incubation with 100 μg/mL of TPGS (the highest concentration used in GKA-NSps for the MTT assay), all of the tested cells retained over 95% viability. Meanwhile, in the 0.1% DMSO/culture medium solution (v/v), which is the highest concentration used in free GKA for the MTT assay, nearly 100% of the tested cell lines remained viable. These results indicated that DMSO used in the experiment had no influence on the cell growth and TPGS can be used as a safe stabilizer.

Among all the test cell lines, MCF-7 breast carcinoma cells seemed most sensitive to GKA-NSps with the lowest IC 50 values, which were used in the subsequent studies. The relatively higher IC50 of normal endothelial cell lines HUVECs 14.82 ± 2.84, Figure 3(A) (b) suggested that GKA-NSps may have a certain selectivity for tumor cells.

### *In vivo* antitumor activity

The *in vivo* antitumor effect of GKA-NSps was investigated using PTX injection as the positive control. The tumor volume growth curves are displayed in Figure 3(B) (a), the body weight change is depicted in Figure 3(B) (b), the average tumor weight and the calculated tumor inhibition rate are shown in Table 3, and the tumors of different groups are shown in Figure S7.

**Table 3.** Inhibiting effect of GKA-NSps in the MCF-7 model (mean ± SD, *n* = 7).

Formulation	Tumor weight (g)	Inhibition rate (%)
GKA-NSPs (20 mg/kg, i.v.)	0.668 ± 0.095**	53.14 ± 6.67
GKA-NSPs (40 mg/kg, i.v.)	0.647 ± 0.091**	54.60 ± 5.45
GKA-NSPs (60mg/kg, i.v.)	0.540 ± 0.106**	62.09 ± 7.45
GKA-NSPs (60 mg/kg, i.g.)	0.819 ± 0.089**,*,#	42.48 ± 6.25
PTX injection (8 mg/kg, i.v.)	0.552 ± 0.133**	61.27 ± 9.34
Free TPGS (60 mg/kg, i.v.)	1.398 ± 0.187	1.83 ± 13.13
Saline	1.424 ± 0.291	–^a^

a The data are not meaningful.

*p < .05 vs. GKA-NSPs (60 mg/kg, i.v.);

**p < .01 vs. Saline;

#*p* < .05 vs. PTX injection (8 mg/kg, i.v.).

As shown in Figure 3(B) (a), the tumor volumes in the normal saline group and free TPGS group were similar, rapidly increased to more than 1300 mm^3^ at the end of experiment, while all mice treated with GKA-NSps showed a significantly smaller tumor volume. Possibly due to the lower bioavailability of oral administration and the absence of the EPR effect, the GKA-NSps (60 mg/kg, i.g.) group displayed the fastest tumor growth among all of the experimental groups, even faster than the 1/3 dose of the GKA-NSps (20 mg/kg, i.v.) group. The tumor inhibition rate data (Table 3) also indicated that GKA-NSps (20 mg/kg, i.v.) had better anti-tumor efficacy than GKA-NSps (60 mg/kg, i.g.) (53.14% vs. 42.28%). This demonstrated that intravenous administration was more effective than oral administration for GKA-NSps to treat tumor. The reasons can be explained as follows. First, the i.v. administration had a much higher bioavailability than the i.g. administration. Second, long-circulation and sustained drug release of GKA-NSps provided more opportunity for nanosuspensions and drug to reach the tumor site. Third, the nanometer particle size helped more GKA-NSps accumulate in tumor via the EPR effect (Jain, 2005; Yuan et al., 2015; Hong et al., 2016; Hong et al., 2017). In addition, GKA-NSps were more potent than free GKA as evidenced in the previous section.

Although GKA-NSps (20 mg/kg, i.v.) and GKA-NSps (40 mg/kg, i.v.) led to a very weak dose-dependent effect according to either tumor volume growth Figure 3(B) (a) or mean tumor weight (Table 3), the high dose of GKA-NSps (60 mg/kg, i.v.) showed an evident dose-dependent effect in contrast with GKA-NSps (40 mg/kg, i.v.). This phenomena may be related to the antitumor mechanism of GKA, such as strengthening of host immunity and reducing the levels of inflammatory cytokine (Wang et al., 2015).

The tumor volume of the high-dose group of GKA-NSps (60 mg/kg, i.v.) was even less than that of the positive control group (8 mg/kg). No significant difference was observed between these two groups according to the tumor inhibition rate (62.1% vs. 61.3%, Table 3). This demonstrated that GKA-NSps (60 mg/kg, i.v.) can be comparable to PTX when used for the treatment of MCF-7 tumor. Possibly due to the very low oral bioavailability (1.1%) (Song et al., 2013), free GKA (bulk GKA powder suspended in water, 60 mg/kg, i.g.) displayed insignificant antitumor efficacy (data not shown), indicating that the antitumor efficacy of GKA can be greatly enhanced using nanotechnology.

Systemic toxicity is a key factor to consider during cancer therapy. Body weight and LD50 are the commonly used indicators of systemic toxicity. There was no body weight loss in normal saline, free TPGS groups, and all GKA-NSps groups Figure 3(B) (b), while the PTX group showed significant body weight reduction after the third dose, indicating the relatively good safety of GKA-NSps. The body weight growth profiles of the three intravenously administered GKA-NSps groups were very similar to that of normal saline. However, there is nearly no body weight growth for the orally administered GKA-NSps (60 mg/kg, i.g.), suggesting better safety of intravenous administration than oral administration for GKA-NSps. This may be because the presence of high dose of GKA-NSps in gastrointestinal tract had a disadvantageous effect on the gastrointestinal functions. This was manifested in the acute toxicity trial (Table S1) that at the highest dose of 320 mg/kg, intravenous administration of GKA-NSps did not lead to death among the tested 10 Kunming mice. This indicated that the minimal lethal dose for GKA-NSps (i.v.) was more than 320 mg/kg, and intravenous administration of GKA-NSps was very safe.

Unlike most chemotherapeutics widely used in clinic, GKA also displayed many beneficial effects on human health, such as anti-inflammatory action, anti-oxidation, neuroprotection, hepatoprotective effect and so on (Suh et al., 1995; Kim et al., 2004; Brozic et al., 2009; Silvan et al., 2011; Gao et al., 2014; Yang et al., 2015), which may have a unique advantage over the current chemotherapeutics in life quality improvement along with tumor suppression. However, the poor solubility, the very low oral bioavailability (approximately 1.1%) (Song et al., 2013) and the weak potency prevented this advantage from becoming reality. These may also be the main reasons why thus far there was no report on systemic *in vivo* antitumor efficacy using GKA alone. The exciting result in our study, such as 62% of TIR and minimal lethal dose of more than 320 mg/kg demonstrated that using nanotechnology, GKA is likely to be developed into a safe and effective anti-tumor drug in clinic.

Nano Chinese medicine is a research field that has attracted significant attention. Especially for the hydrophobic active compounds or fractions from Chinese medicine and natural resources, nanotechnology has proven to be an effective strategy to deal with insolubility and the resultant low bioavailability and difficulty of drug delivery. GKA represents a large type of flavonoids that have extensive but weak biological activity. The data in this paper demonstrated that nanotechnology can help resolve the abovementioned issues related to poor solubility but also significantly improve their druggability and promote their clinical application.

## Conclusions

The major problem for the chemotherapeutics currently used in clinic is the serious side effects. Thus, the discovery of safe antitumor agents with less adverse effects becomes one of the emphases of antitumor therapy. GKA, a type of low toxicity and widely available flavonoids, has extensive beneficial effect on human health and has certain anti-tumor effects. However, the poor solubility and oral bioavailability greatly limited the further *in vivo* study. In this paper, GKA was formulated into nanosuspensions with a high drug loading content of nearly 50%, which successfully solved the difficulties of poor solubility. The resultant GKA-NSps had a size of approximately 180 nm using TPGS as a stabilizer and effectively improved *in vitro* antitumor activity against many tumor cell lines in contrast with free GKA. The resultant GKA-NSps also showed good stability in various physiological media with no hemolysis and sustained drug release, which met the requirements of intravenous injection, and could improve bioavailability. The *in vivo* study demonstrated intravenous administration of GKA-NSps, which achieved a tumor inhibition rate of 62%, while the minimal lethal dose was more than 320 mg/kg. GKA-NSps showed effective anti-tumor effect and good tolerance and were likely to become a safe and effective antitumor drug in the future. To our knowledge, this is the first time that GKA was prepared into nanoparticles and used for *in vivo* antitumor therapy with an exciting efficacy. Therefore, GKA-NSps in this study with a small size, high drug loading content, and significant anti-tumor activity have a good application prospect in anti-tumor research. The approach used here may also be applicable to other antitumor flavonoids for R&D of new safe antitumor drugs.

## Supplementary Material

IDRD_Wang_et_al_Supplemental_Content.docx
