# Supplementary material for: Genkwanin nanosuspensions: a novel and potential antitumor drug in breast carcinoma therapy
Source: Drug Deliv. 2017 Sep 29;24(1):1491–500. doi: 10.1080/10717544.2017.1384519 (PMC8241161; doi:10.1080/10717544.2017.1384519)
Supplement: IDRD_Wang_et_al_Supplemental_Content.docx [file IDRD_A_1384519_SM5226.docx]

**·Appendices**

**Table S1 Kunming (KM) mice survival situation during the 14-d observation after a single dose of GKA-NSps (i.v., n=10)**

| Dose (mg/kg) | Survival rate of KM mice (%) | |
| --- | --- | --- |
|  | female | males |
| 120 | 100% | 100% |
| 160 | 100% | 100% |
| 200 | 100% | 100% |
| 240 | 100% | 100% |
| 280 | 100% | 100% |
| 320 | 100% | 100% |


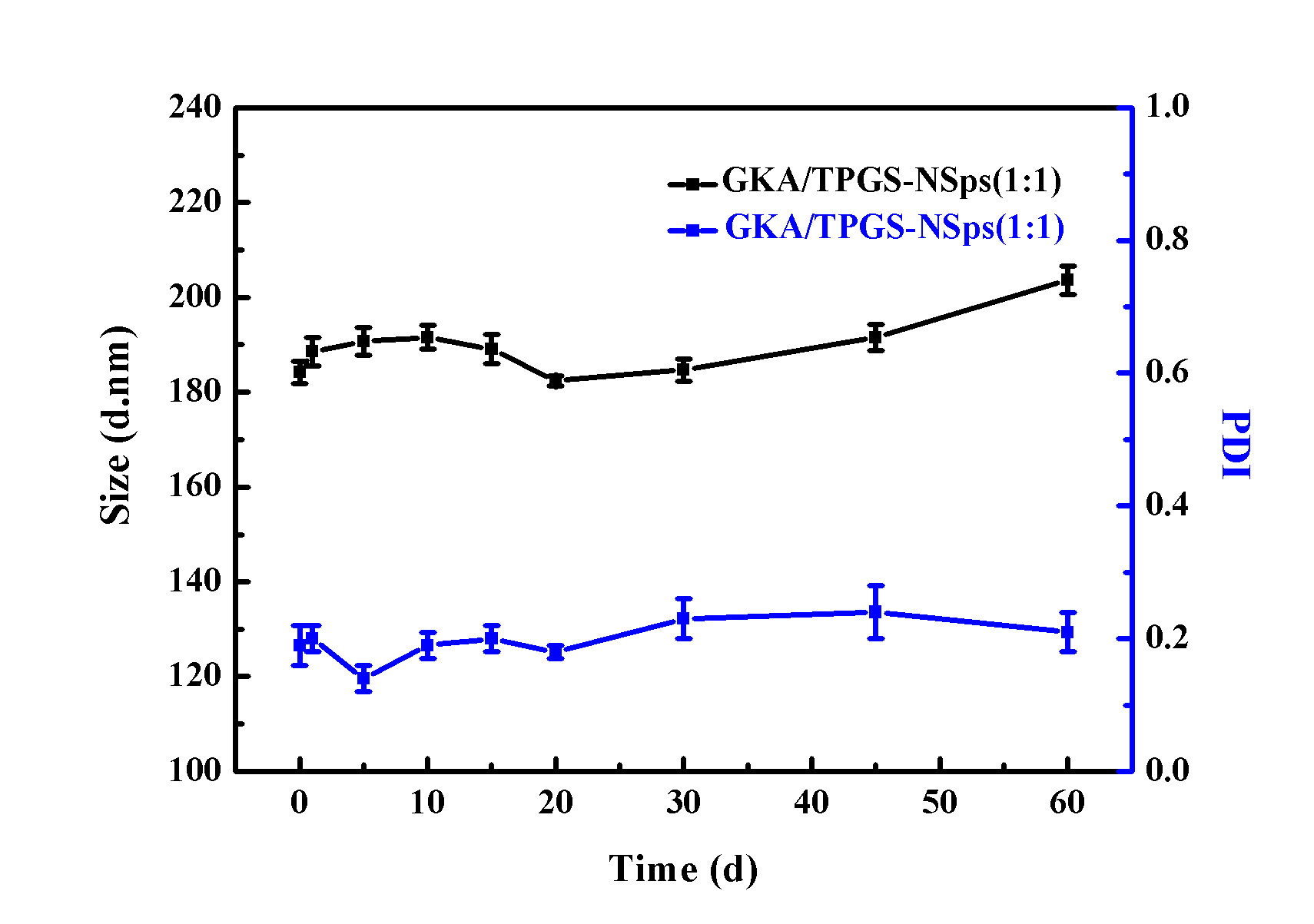

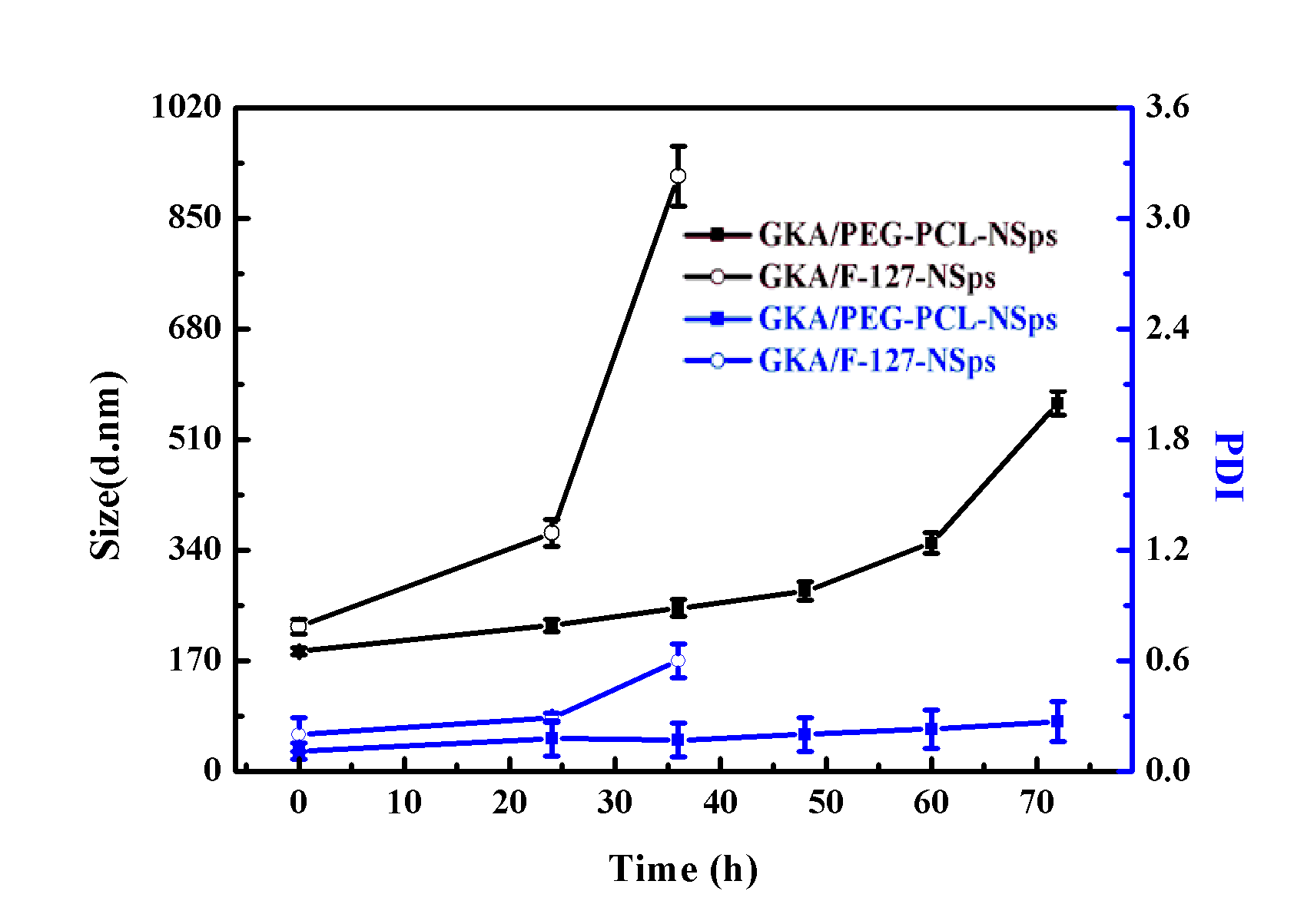


**Figure S1.** Size and PDI of GKA-NSps prepared using different stabilizers during storage at 4°C.


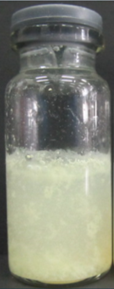


**Figure S2.** Photograph of free GKA in water


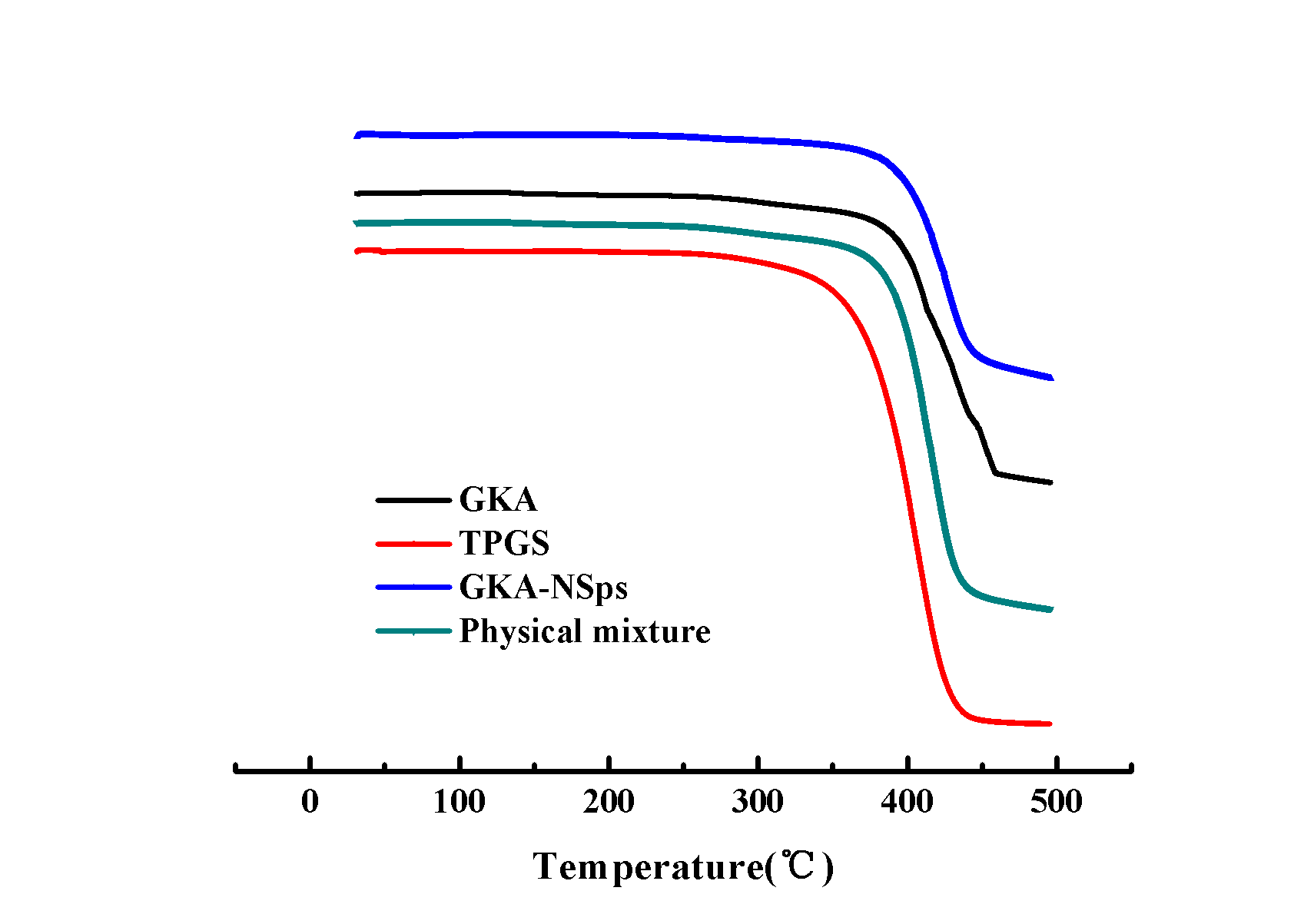


Figure S3. Thermogravimetric analysis pattern of GKA bulk powder, stabilizer (TPGS), GKA-NSps, and the physical mixture of GKA bulk powder and TPGS.





**Figure S4.** Hemolysis percentage of GKA-NSps at different concentrations (mean ± SD, n = 3).


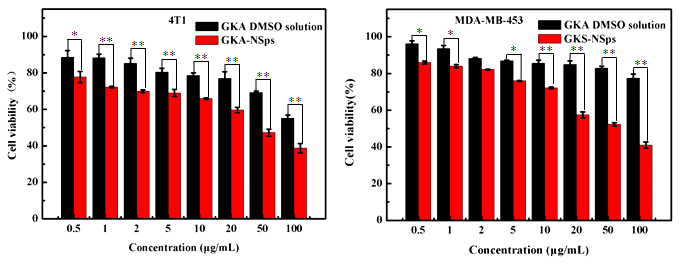


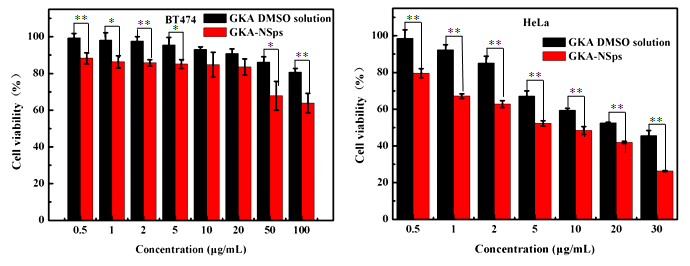


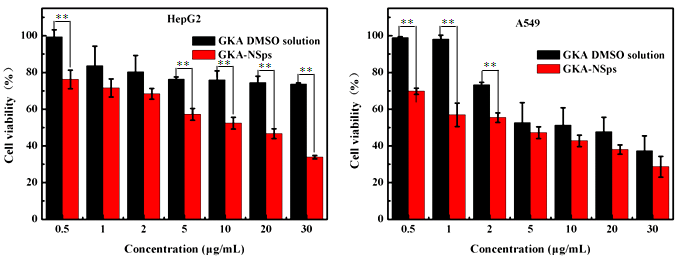


**Figure S5.** Cytotoxicity of GKA-NSps and GKA DMSO solution against different cancer cells after 48 h of incubation (mean ± SD, *P<0.05, **P<0.01).


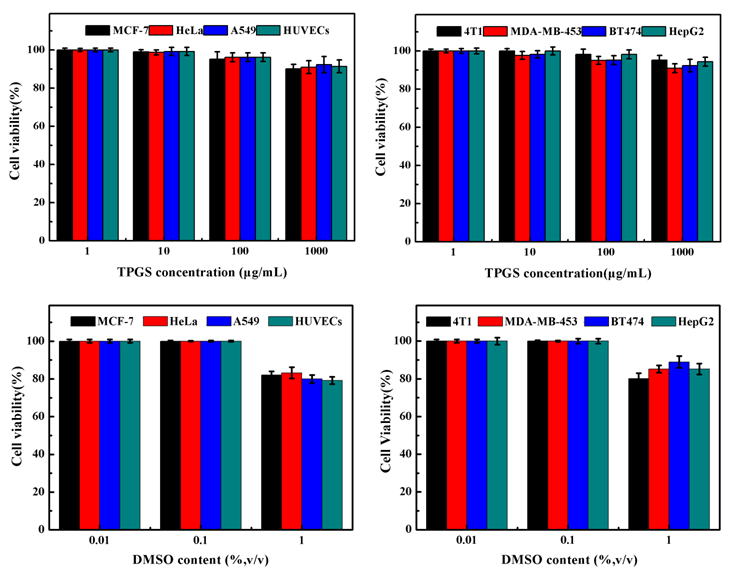


**Figure S6.** Cytotoxicity of TPGS and DMSO against different cells after 48 h of incubation (mean ± SD).

**
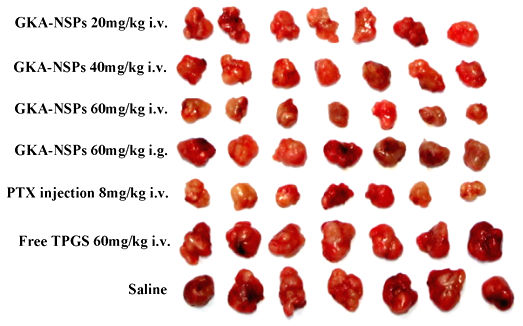
**

**Figure S7. Photo of tumors collected from the tested mice at the end of the experiment.**
